# eIF4E promotes tumorigenesis and modulates chemosensitivity to cisplatin in esophageal squamous cell carcinoma

**DOI:** 10.18632/oncotarget.11694

**Published:** 2016-08-30

**Authors:** Ting Liu, Rong Li, Hui Zhao, Juan Deng, Ying Long, Meng-ting Shuai, Qian Li, Huan Gu, Ya-qi Chen, Ai-min Leng

**Affiliations:** ^1^ Department of Gastroenterology, Xiangya Hospital of Central South University, Changsha, Hunan, China; ^2^ Department of Gastroenterology, Third Xiangya Hospital of Central South University, Changsha, Hunan, China

**Keywords:** esophageal squamous cell carcinoma, eIF4E, cisplatin, chemosensitivity, PI3K/AKT pathway

## Abstract

Patients with esophageal squamous cell cancer are often diagnosed with advanced diseases that respond poorly to chemotherapy. Overexpression of eIF4E leads to enhance the translation of key malignancy-related proteins and enabling tumor growth and chemoresistance in a variety of human malignancies, but whether it has a role in ESCC remains obscure. We hypothesized that eIF4E promoted ESCC tumorigenesis and facilitated the development of acquired resistance to the cisplatin-based chemotherapy. In this study, we showed that eIF4E expression was increased significantly in clinical ESCC tissues and and ESCC cell lines and its expression level was correlated with lymph node metastasis, TNM stage, as well as overall and disease-free survival of ESCC. We also showed here that knockdown of eIF4E in EC9706 would dramatically reduced cell proliferation, colony formation, migration and invasion, apoptosis *in vitro* as well as *in vivo*, and vice versa. Moreover, “weak mRNAs” were demonstrated to be regulated by eIF4E in ESCC, which might interpret the above function. Overexpression of eIF4E decreased the efficacy of cisplatin-induced cell growth inhibition in ESCC cell line and xenograft model (*P* < 0.05). eIF4E knockdown by shRNA increased cisplatin-induced cytotoxicity in ESCC cell lines, and enhanced chemosensitivity to cisplatin in xenograft tumor models. Furthermore, we found that the PI3K/AKT pathway and Bcl-2/Bax ratio might be responsible for the eIF4E-induced cisplatin resistance in ESCC. Our data collectively show association of eIF4E expression with chemotherapeutic response in ESCC, and suggest that therapeutically targeting eIF4E may be a viable means of improving chemotherapy response in ESCC.

## INTRODUCTION

The incidence of esophageal cancer ranks eighth in malignant diseases all over the world, and the mortality rate ranks the sixth [1]. Squamous cell carcinoma and adenocarcinoma are two major types of esophageal cancer. Over 90% cases are esophageal squamous cell carcinoma (ESCC) in a high incidence areas called esophageal “cancer belt”, which extends from the Middle East to Northeast China. ESCC is often tightly associated with extensive lymphatic spread and vascular invasion, and early symptoms are usually absent. Diagnosis most commonly occurs in the later stages of the disease thereby decreasing the survival rate. Neoadjuvant chemotherapy followed by surgery has become a promising strategy for advanced esophageal cancer. However, the reported response rate to cisplatin-based chemotherapy is only modest [2]. Therefore, it is important to identify and target genes beneficial to the treatment of ESCC, such as enhancement of conventional chemotherapy and improving the ability to predict the response to chemotherapy before treatment.

Translation is a multistep, multifactorial process consisting of 3 major parts: initiation, elongation and termination. Among them the initiation is the most complex and the most tightly controlled, which is an essential preparation stage for the ribosome to position on the mRNA to be translated correctly, then commence the extension. A set of proteins named translation initiation family (eIFs, eukaryotic initiation factors) involved in the translation initiation in mammalian cells [3]. They are vital and of the essence to modulate the initiation, blockade or stimulation of these proteins will inhibit or induce mRNA expression [4]. Since eIF4E is the least abundant but the most important member in the family, its recruitment serves as the rate limiting step in the initiation of cap dependent translation.

Cellular mRNAs differ greatly in the amount of eIF4E they require for efficient translation and in the composition of their 5′untranslated regions (5′UTRs). It has been proposed that the mRNA 5′UTRs structure in part dictates translation efficiency. mRNAs with highly complex 5′UTR structures named weak mRNAs are more difficult to be translated than those with relatively uncomplicated structures called “strong” mRNAs [5]. Under normal circumstances, eIF4E is typically sequestered by hypophosphorylated 4E-BPs, resulting in restricted translation rates. Homeostasis is maintained by limiting translation to essential genes (such as housekeeping genes), and weak mRNAs (several proto-oncogenes and angiogenic factors) translation is restrained [6, 7]. Weak mRNAs including C-myc, Cyclin D1, Pim-1, Survivin, Bcl-2, VEGF, FGF-2 and MMP-9, are regarded as onco-proteins, pro-survival proteins, angiogenesis factors and proteins involved in oncogensis, tumor invasion and metastasis. These weak mRNAs require a higher dependency on eIF4E for translation initiation. They have been demonstrated to be excessively activated by increased eIF4E [5, 8–10]. Even small changes in eIF4E levels are believed to broadly impact mRNA translation, as well as cellular and organismal function [11, 12]. That will promote the cell step into the next cycle and proliferate, thus initiate oncogenesis and promote tumor progression [13].

Malignant tumor formation is well known as a multi-stages complex process with multi-genes involved in and multi-factors coordinated. Proliferation of malignant tissue needs variable proteins continual synthesis, thus over-expression of eIF4E is an inevitable event. Recent studies have revealed that excessive eIF4E could change cell phenotype and participate in the induction of cell proliferation, cell transformation and tumorigenesis, invasion and metastasis [10, 14–17]. Excessive expression of eIF4E is significantly correlated with unfavorable clinical outcomes such as pathological grading of tumor, high cellular proliferation, and poor prognosis in several cancers. Importantly, eIF4E has been regarded as a novel target to synergize drug efficiency through multiple pathways intervention [10, 14, 18, 19].

Although the expression of eukaryotic initiation factors (eIFs) including eIF4E in several tumors and their role in chemoresistance have been previously reported [18, 19], the role of eIF4E in oncogenesis and drug resistance in ESCC, however, remains unclear. In the present work, we studied the expression pattern of eIF4E in ESCC cell lines and clinical ESCC tissues and explored the potential oncogenic role of eIF4E in ESCC. We also examined the association between eIF4E expression and cisplatin-based chemotherapy response in ESCC cell lines and xenograft models, and explored its potential mechanism.

## RESULTS

### eIF4E overexpressed in ESCC and reduced patient survival

**Figure 1:**
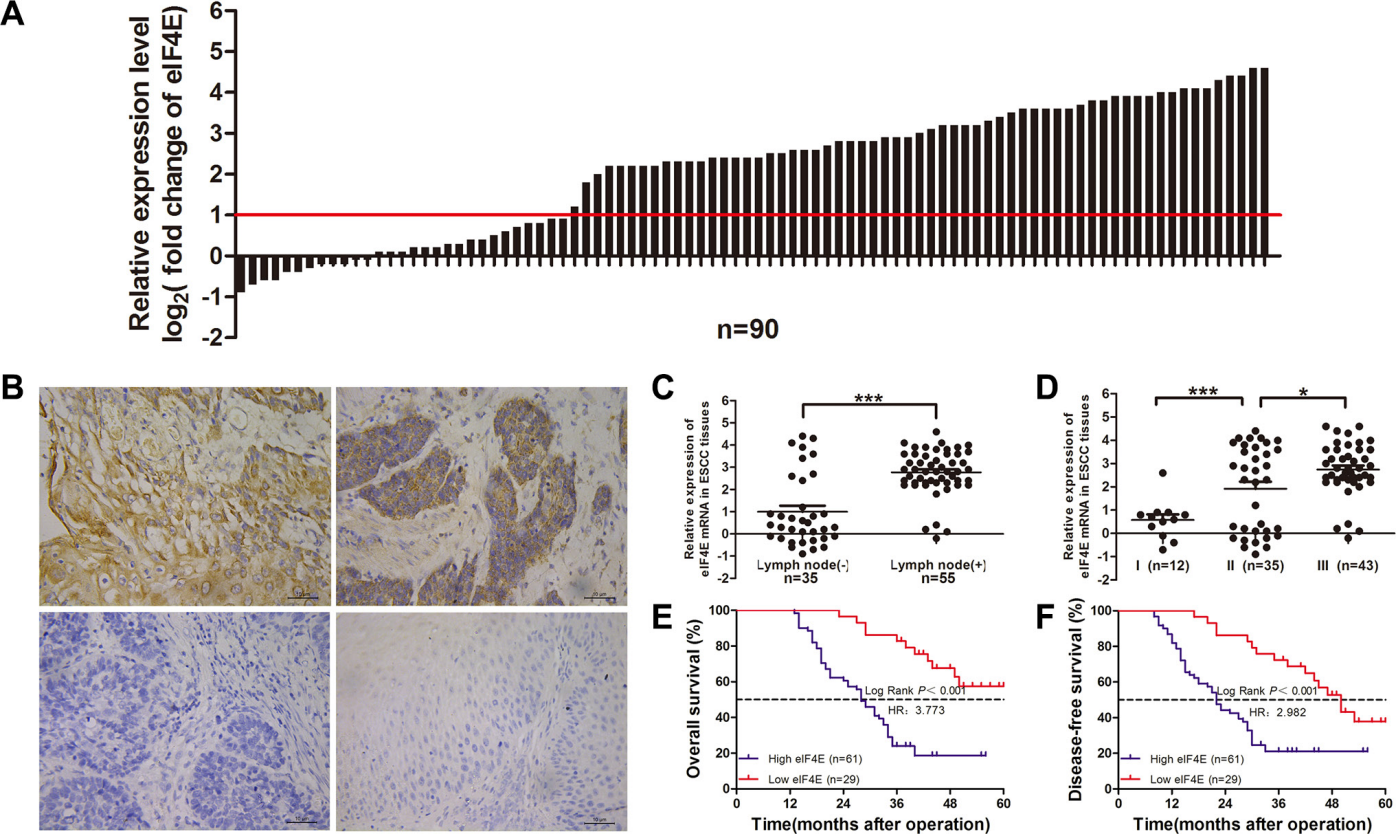
Overexpression of eIF4E in clinical ESCC tissues and the association with survival outcome in ESCC patients (**A**) Relative eIF4E mRNA expression level (log_2_ fold change) was measured by qRT-PCR in 90 paired ESCC tissues; (**B**) Different eIF4E expression level was measured by IHC staining, upper left) high eIF4E expression, upper right) moderate eIF4E expression, low left) no eIF4E expression exhibited in ESCC tissues, low right) little eIF4E expression was found in normal esophageal tissues (X400). The ESCC patients were classified into low eIF4E expression group and high eIF4E expression group according to the relative eIF4E mRNA expression level. The high expression of eIF4E (*n* = 61) was highly related to lymphonodus involvement (**C**, *P* < 0.001) and TNM staging (**D**, I *vs*. II *P* = 0.0004; II *vs*. III, *P* = 0.145). Kaplan-Meier survival analysis of eIF4E expression in ESCCs: the patient with high expression of eIF4E has a much shorter OS (*P* < 0.001) (**E**) and DFS (*P* < 0.001) after operation (**F**).

We evaluated eIF4E RNA and protein expression by RT-qPCR and immunohistochemistry in 90 paired ESCC tissues. Most ESCC tissues showed a significant high eIF4E mRNA expression (Figure 1A, 61.11% vs. 38.89%, *P* < 0.05) when compared to adjacent non-cancerous tissues (ANCTs), eIF4E immunostaining was scored for percent area stained and immunostaining intensity. All ESCC tumors, ANCTs or normal esophageal tissues, showed similar percent area stained for cytoplasmic eIF4E but varied in staining intensity. eIF4E protein was stained as yellow or brown substances that mainly existed in the cytoplasm or around the nucleus. Three examples shown in Figure 1B represent some extremes, from strongest to intermediate and no staining of eIF4E in ESCC tissues. According to IHC analysis, eIF4E protein expressed extensively in tumor tissues (81.11%, 73/90), while only 22.22% (20/90) in ANCTs and 16.67% (6/36) in normal esophageal tissues (Control group). Moreover, a positive relationship between the eIF4E mRNA expression and the eIF4E protein was found (Supplementary Figure S1). These data show that eIF4E expression is significantly increased across all ESCC tissues.

To further evaluate the role of eIF4E in human ESCC, we next examined the association between eIF4E and several clinical parameters, including age, gender, TNM, and cancer grade in 90 ESCC patients. According to discribed before, patients were segregated into high and low eIF4E expression groups. Clinical characteristics of patients were listed in Table 1. The chi-square test showed high eIF4E expression was significantly related to the larger lesion (*P* = 0.042, Table 1), the lymphonodus involvement (*P* < 0.001, Table 1 and Figure 1C) and TNM stage (*P* < 0.001, Table 1 and Figure 1D). Similar to previous report [21], eIF4E did not correlate with other clinical and pathologic characteristics, including age (*P* = 0.326), gender (*P* = 0.769).

**Table 1:** eIF4E Expression Level and Clinicopathological Characteristics in 90 Cases of ESCC

Clinicopathological Variables	*n*	eIF4E Expression		*P*
		low	high	
Age (years)				0.326
≤ 60	63	18	45	
> 60	27	11	16	
Gender				0.769
Female	16	6	10	
Male	74	23	51	
Tumor size (cm)				**0.042**
≤ 4	44	19	25	
> 4	46	10	36	
Differentiation				0.416
Well	32	13	19	
Moderate	49	13	36	
Poor	9	3	6	
T Stage				0.069
T1	5	4	1	
T2	37	13	24	
T3	40	11	29	
T4	8	1	7	
N Stage				**< 0.001**
N0	40	26	14	
N1	28	2	26	
N2	22	1	21	
TNM Stage				**< 0.001**
I	17	11	6	
II	39	17	22	
III	34	1	33	

To investigate whether overexpression of eIF4E is correlated to unfavorable outcome in ESCC patients, we analyzed the association of eIF4E expression level with other well-known clinical parameters using Cox proportional hazards regression model and multivariate survival analysis (Table 2). Kaplan-Meier indicated that overall survival time was significantly dependent on eIF4E expression levels. Patients in the high eIF4E expression group were ~3.7 times likelier to die of ESCC than patients with low eIF4E levels (median survival time: 28 months versus > 60 months, *P* < 0.001; Figure 1E). Similarly, the disease-free survival time in high eIF4E expression patients were ~2.9 times shorter than patients with low eIF4E expression (median survival time: 22 months versus 50 months, *P* < 0.001; Figure 1F). Moreover, multiple COX analysis in Table 3 demonstrated that eIF4E along with N stage, TNM stage were independent indicator for ESCC prognosis.

**Table 2:** Univariate cox analysis of overall and disease-free survival in 90 patients with ESCC

Variables	*n*	Overall Survival		Disease-Free	Survival
		HR (95% CI)	*P*	HR (95% CI)	*P*
Age (years)					
< 60	63	1		1	
≥ 60	27	1.287 (0.725–2.283)	0.389	1.375 (0.795–2.378)	0.254
Gender					
Female	16	1		1	
Male	74	0.971 (0.488–1.929)	0.932	0.923 (0.479–1.778)	0.810
Tumor size (cm)					
≤ 4	44	1		1	
> 4	46	1.649 (0.964–2.821)	0.068	1.548 (0.924–2.592)	0.097
Differentiation			0.158		0.191
Well	32	1		1	
Moderate	49	1.714 (0.933–3.151)	0.083	1.664 (0.933–2.969)	0.085
Poor	9	2.080 (0.804–5.379)	0.131	1.815 (0.714–4.616	0.211
T Classification			**< 0.001**		**< 0.001**
T1	5	1		1	
T2	37	0.485 (0.065–3.645)	0.482	0.477 (0.064–3.580)	0.472
T3	40	1.130 (0.153–8.349)	0.905	1.154 (0.157–8.498)	0.888
T4	8	4.776 (0.582–39.202)	0.145	5.890 (0.715–48.531)	0.099
N Classification			**< 0.001**		**< 0.001**
N0	40	1		1	
N1	28	3.113 (1.542–6.282)	**0.002**	2.864 (1.482–5.532)	**0.002**
N2	22	14.401 (6.770–30.634)	**< 0.001**	14.948 (7.105–31.449)	**< 0.001**
TNM Stage			**< 0.001**		**< 0.001**
I	17	1		1	
II	39	5.325 (1.245–22.773)	**0.024**	4.169 (1.249–13.909)	**0.020**
III	34	33.569 (7.810–144.287)	**< 0.001**	26.226 (7.762–88.606)	**< 0.001**
eIF4E expression					
Low	44	1		1	
High	46	6.080 (2.725–13.568)	**< 0.001**	4.686 (2.343–9.369)	**< 0.001**

**Table 3:** Multivariate Cox regression analysis of overall and disease-free survival in 90 Patients with ESCC

Variables	*n*	Overall Survival		Disease-Free	Survival
		HR (95% CI)	*P*	HR (95% CI)	*P*
T Classification			0.082		**0.032**
T1	5	1		1	
T2	37	0.117 (0.007–1.934)	0.134	0.171 (0.015–1.978)	0.157
T3	40	0.127 (0.007–2.346)	0.165	0.194 (0.015–2.538)	0.211
T4	8	0.306 (0.015–6.215)	0.441	0.580 (0.039–8.532)	0.691
N Classification			0.054		**0.025**
N0	40	1		1	
N1	28	0.681 (0.212–2.187)	0.518	0.707 (0.220–2.267)	0.560
N2	22	1.688 (0.372–7.649)	0.497	1.996 (0.435–9.153)	0.374
TNM Stage			**0.040**		**0.042**
I	17	1		1	
II	39	9.169 (1.084–77.585)	**0.042**	5.508 (1.096–27.674)	**0.038**
III	34	24.133 (1.722–338.274)	**0.018**	14.734 (1.567–138.542)	**0.019**
eIF4E expression					
Low	44	1		1	
High	46	3.600 (1.365–9.498)	**0.010**	2.784 (1.167–6.640)	**0.021**

### Overexpressed eIF4E promoted cell proliferation, colony formation, migration and invasion, anti-apoptosis

**Figure 2:**
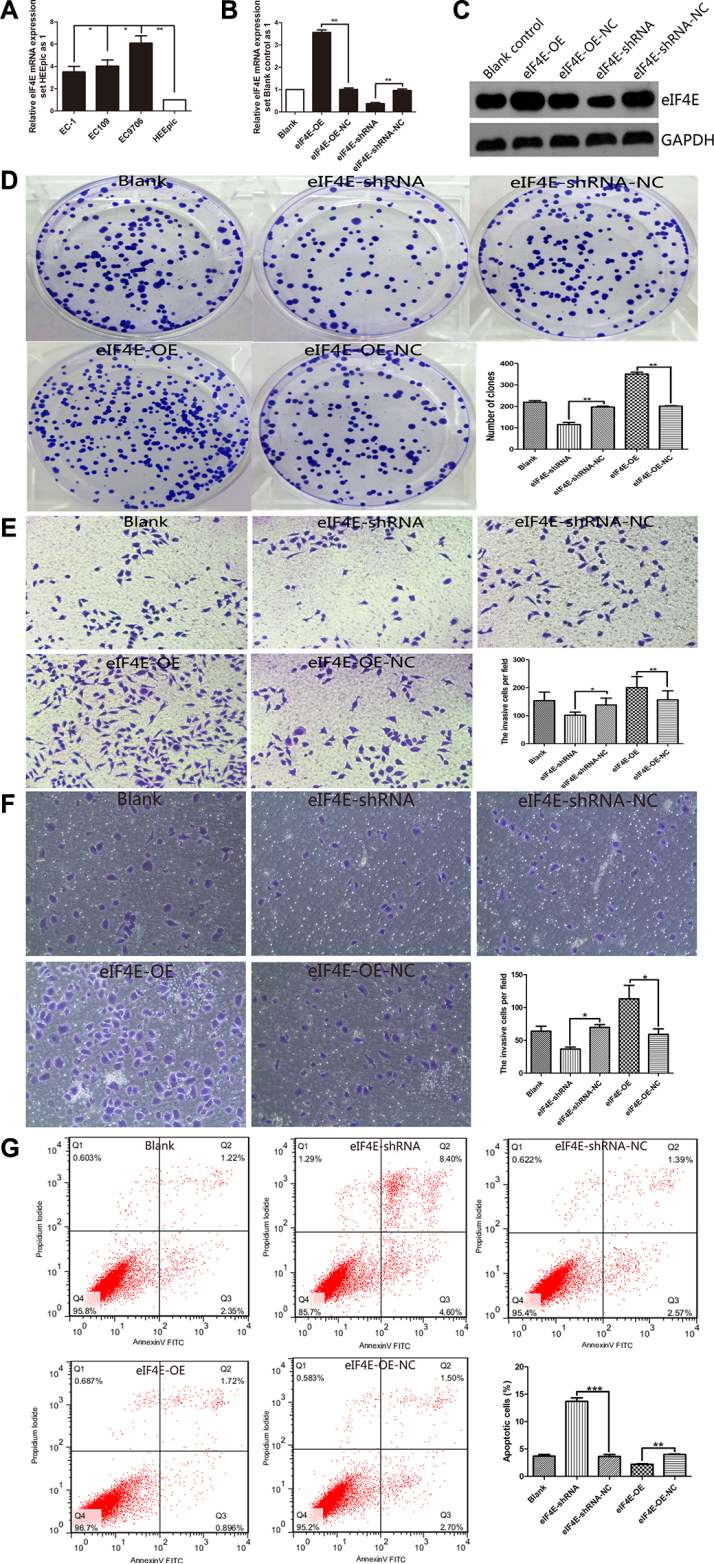
eIF4E promote proliferation, migration and invasion, anti-apoptosis in ESCC cell (**A**) The level of eIF4E was increased in ESCC cell lines including EC-1, EC109 and EC9706, when compared to the normal Human Esophageal Epithelial Cells *HEEpic.* EC9706 has the highest eIF4E expression among these 3 ESCC cell lines. (**B**, **C**) EC9706 cells have be transfected with eIF4E-PEGFP-N1 for eIF4E-overexpression (eIF4E-OE) and with eIF4E-shRNA for eIF4E-knowdown. The control cells are transfected with eIF4E-OE-NC or eIF4E-shRNA-NC. The effect of eIF4E overexpression (*P* < 0.01) and knockdown (*P* < 0.01) was confirmed with qPCR and Western blotting, respectively. (**D**) Down-regulation of eIF4E suppressed colony formation compared with negative controls, while promoted colony formation after eIF4E-OE. The number of colonies were calculated and depicted by the ban graph. (**E**, **F**) The number of migrating or invading cells in the eIF4E-shRNA group was significantly decreased compared with the negative control, but increased in the eIF4E-OE group. (**G**) Apoptotic rate was detected by flow cytometry assay with double staining by Annexin V-FITC/Propidium Iodide. Overexpressed eIF4E decreased the apoptotic rate including early apoptosis and late apoptosis. Inhibition of eIF4E promoted apoptosis in EC9706 cells. Data are represented as the mean ± SD of three independent experiments. Error bars indicate s.d. (*n* = 3). *P < 0.05, **P < 0.01.

In order to clarify the role of eIF4E involved in ESCC oncogenesis, we investigated the potential effect of eIF4E on proliferation, migration and invasion, apoptosis in ESCC cells. According to eIF4E expression in esophageal cell lines, qPCR showed that eIF4E was highly expressed in all three cancer cell lines (*P* < 0.05 for *EC-1* & *EC109* vs. *HEEpic*, and *P* < 0.01 for *EC9706* vs. *HEEpic*) and EC9706 has the highest eIF4E expression (*P* < 0.05 for *EC9706 vs. EC-1* or *EC109*) among these 3 ESCC cell lines, whereas it was weakly detected in Human Esophageal Epithelial Cells *HEEpic* (Figure 2A). We then selected EC9706 cells to be transfected with eIF4E-PEGFP-N1 for eIF4E-overexpression (eIF4E-OE) and with eIF4E-shRNA for eIF4E-knowdown. The cells transfected with eIF4E-PEGFP-N1-NC (eIF4E-OE-NC) or eIF4E-shRNA-NC are used as controls respectively. The effect of eIF4E overexpression and knockdown was confirmed with qPCR (Figure 2B) and Western blotting (Figure 2C).

To investigate the impact of eIF4E on cell proliferation, MTT analysis and colony formation assays were conducted. The data showed that up-regulation of eIF4E increased the proliferation of EC9706 cells (Supplementary Figure S2A), and vice versa. Similarly, colony formation assays showed that the ability of proliferation in EC9706 cells were significantly repressed by down-regulation of eIF4E (Figure 2D). Furthermore, we found that overexpression of eIF4E promoted tumorigenicity *in vivo* when compared to blank control mice (*P* = 0.045, Supplementary Figure S2B), while knockdown of eIF4E significantly decreased the primary tumor size (*P* = 0.032, Supplementary Figure S2B). It indicated a oncogenic role of eIF4E in promoting the tumorgenesis of ESCC.

To assess the effects of eIF4E on migration and invasion, transwell migration and matrigel invasion assays were performed. Figure 2E showed that compared to the negative control, down-regulation of eIF4E could effectively repress the migration ability and invasion capacity of EC9706 cells, while overexpressed eIF4E restored its migration and invasion ability.

Apoptosis analysis showed that there was significant change of apoptotic rate after treating with eIF4E-OE or eIF4E-shRNA (Figure 2F). Overexpressed eIF4E inhibited apoptosis, while promoted apoptosis significantly after eIF4E inhibition, including early apoptosis and late apoptosis (Supplementary Figure S3).

### “Weak mRNAs” were up-regulated by increased eIF4E and inhibited by knock-down of eIF4E

**Figure 3:**
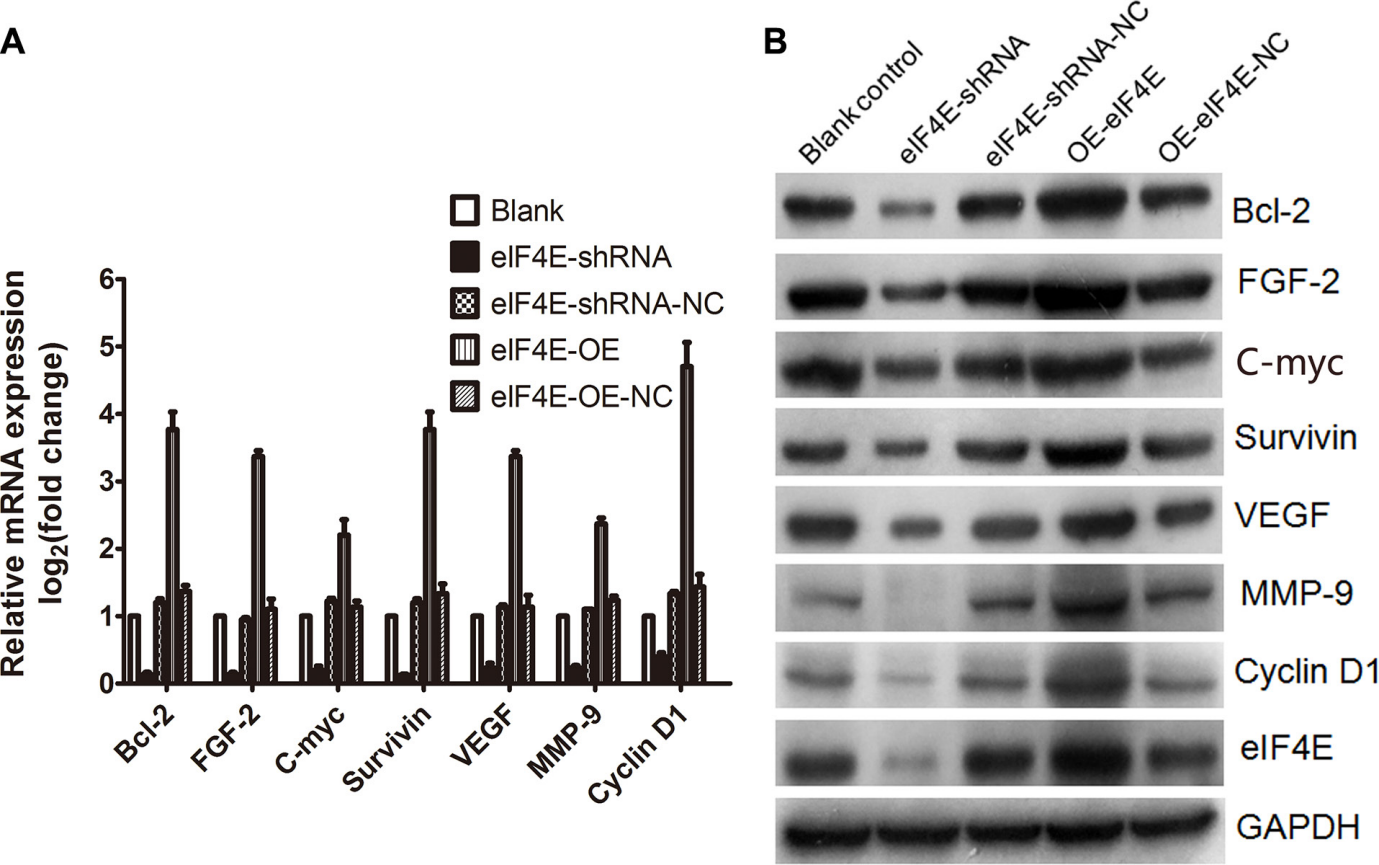
Weak mRNAs was regulated by eIF4E in ESCC cells (**A**) Weak mRNAs, including *C-myc, Cyclin D1, Bcl-2, Survivin, FGF-2, VEGF,* and *MMP-9*, were down-regulated after eIF4E knockdown, but up-regulated when eIF4E was overexpressed. (**B**) Western blotting showed that the translation of “weak mRNAs” were inhibited when eIF4E was knocked down, but promoted when eIF4E was increased. Error bars indicate s.d. (*n* = 3).

Cellular mRNAs must compete to gain combination with eIF4E and translation initiation. Strong mRNAs get preferentially translated, while the weak mRNA are restrained normally. The weak mRNA mostly belong to typical growth factor and proto-oncogene mRNAs. Molecule that antagonise eIF4E can inhibit cellular expression of oncogenic proteins encoded by weak mRNAs [7]. As these weak mRNAs almost universally encode growth regulatory proteins, including C-myc, Cyclin D1, Bcl-2, Survivin, FGF-2, VEGF, and MMP-9 etc [5, 10]. They were reported to promote the tumor cells resistance to apoptosis, proliferation, invasion and distant metastasis, even drug resistance induced by increased eIF4E [10, 14–17]. The present study have clarified the oncogenic role of eIF4E in ESCC, and confirmed that the weak mRNAs including C-myc, Cyclin D1, Bcl-2, Survivin, FGF-2, VEGF, and MMP-9 were regulated by eIF4E in ESCC cell line. As is shown in Figure 3A, they were upregulated after eIF4E was increased in EC9706, while inhibited when the eIF4E was supressed. Figure 3B demonstrated that the increased eIF4E promoted the translation of these target genes. To our knowledge, it was the first report to examine the regulation of weak mRNAs by eIF4E in esophageal cancer cells, which can explain the influence of eIF4E on the oncogenic biology of ESCC.

### Knockdown of eIF4E increased the sensitivity of ESCC to cisplatin *in vitro* and *vivo*

**Figure 4:**
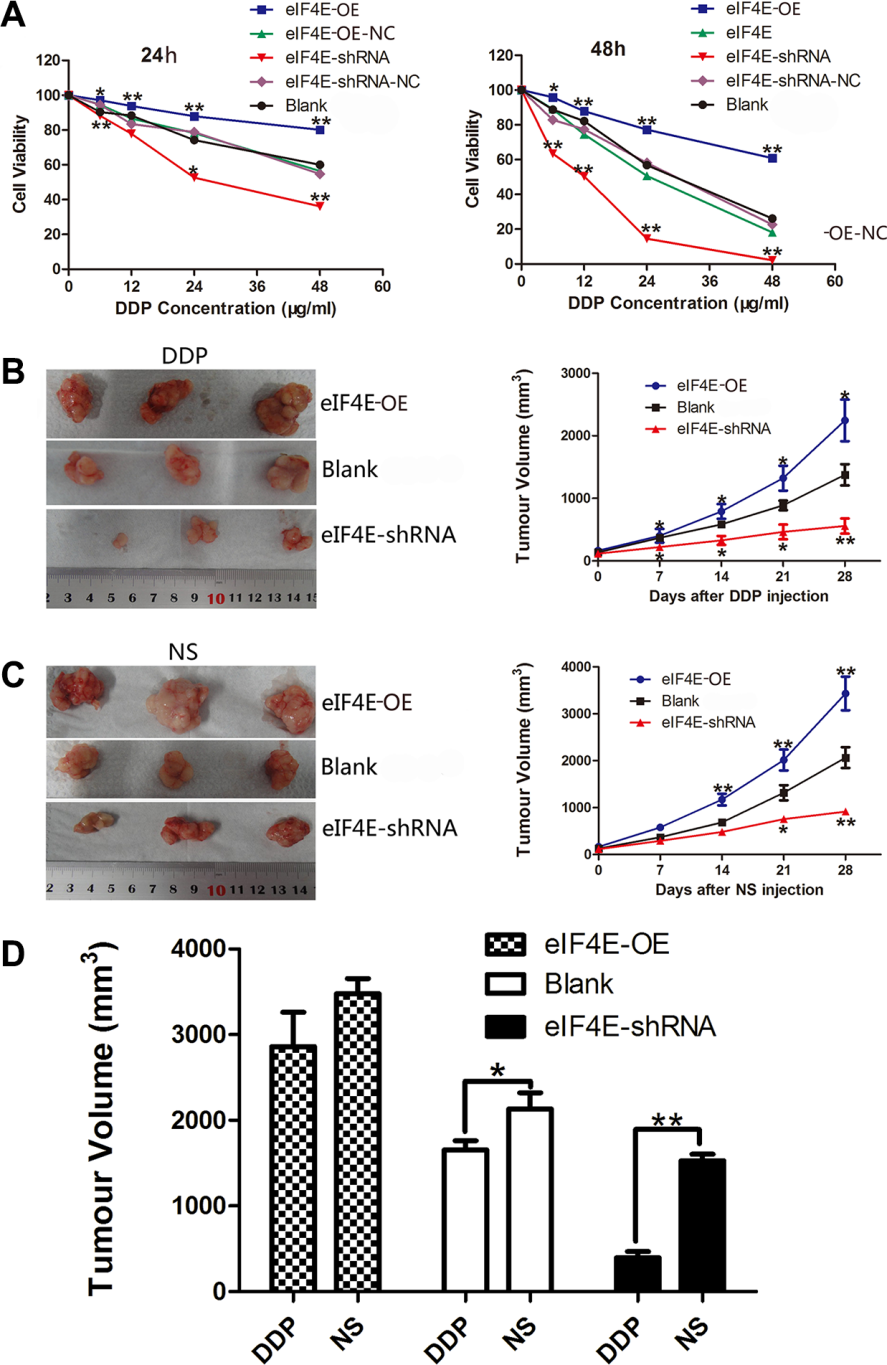
eIF4E knockdown enhanced the chemosensitivity of ESCC to cisplatin *in vitro* and *vivo* (**A**) The cell viability of transfected EC9706 cells with various eIF4E expression and treated with DDP (cisplatin, 0–48 μg/ml) for 24 h and 48 h. The plots show that inhibition of eIF4E greatly impeded cell survival in a time and concentration dependent manner, while elevated eIF4E promoted drug resistance in EC9706 cells. There is no significant difference between the two scramble controls and blank control. (**B**, **C**) Representative macroscopical view of fresh tumor tissues and the dynamic tumor growth curves: eIF4E overexpression contributed to slowing down the rate of cisplatin induced tumor inhibition, and eIF4E knockdown reversed this tendency. The average tumor volume in eIF4E overexpression group was larger than those mice treated with EC9706 only, while the tumor volume in eIF4E knockdown group was significantly smaller than that in the blank control group after DDP or NS injection. (**D**) Comparison of tumor inhibition after 28 days of DDP and NS injection: Tumor in the blank group exhibited a resistance to cisplatin (DDP group *vs*. NS group, *P* = 0.0852). eIF4E overexpression invalidated inhibition efficiency of DDP (DDP group *vs.* NS group, *P* = 0.3034), while eIF4E knockdown boosted the effect of DDP induced tumor inhibition (DDP group *vs.* NS group, *P* = 0.0045). Error bars indicate s.d. (*n* = 3). *P < 0.05; **P < 0.01.

Cisplatin (DDP), 5-fluorouracil (FU), and docetaxel (TAX) are clinically used in neoadjuvant or adjuvant chemotherapy for ESCC. As an effective broad spectrum anti-cancer drug, cisplatin exerts its cytotoxicity by DNA damage to attack cancer cells, while resistance to cisplatin remains a major problem in the clinical treatment of ESCC. To assess whether there is an association between eIF4E expression and chemotherapeutic response in ESCC patients, we next performed an escalating-dose experiment in EC9706 cells to examine the role of eIF4E in tumor cell chemosensitivity, according to the chemosensitivity to DDP in different ESCC cell lines (Supplementary Figure S4). The transfected cells with eIF4E overexpression or knockdown were treated with DDP as described above. MTT cell viability assay showed dose-dependent inhibition of cell proliferation on the drug treatments. In cells treated with DDP, eIF4E knockdown lead to a statistically significant decrease in growth compared to control shRNA treated cells (*P* < 0.01 for comparisons of eIF4E-shRNA to control eIF4E-shRNA-NC), while the cells with eIF4E overexpression have significant superior cell viability (*P* < 0.01 for all comparisons of eIF4E-OE to control eIF4E-OE-NC) (Figure 4A).

In the next set of experiments, we tested whether the knockdown of eIF4E increased chemosensitivity *in vivo*. To this end, EC9706 cells with eIF4E overexpression (transfected with eIF4E-OE) and knockdown (transfected with eIF4E-shRNA) were injected subcutaneously into flanks of nude mice to develop xenograft tumors. Cisplatin (2 mg/kg) was intraperitoneal administered into tumor-bearing mice twice a week for 4 weeks after 10 days of inoculation and mean subcutaneous tumor volume reached 100–200 mm^3^. The antitumor efficacy was measured by monitoring the tumor volume after treatment. Similar to the effects we observed *in vitro*, tumor growth decreased dramatically in eIF4E-shRNA group with DDP treated (Figure 4B, 4C). After treatment of 28 days, the average tumor volume of eIF4E-shRNA group (542.81 ± 98.13 mm^3^, 1399.71 ± 110.24 mm^3^ in DDP and NS, respectively) was significantly smaller than its corresponding blank control (1753.24 ± 115.27 mm^3^, 2163.41 ± 244.45 mm^3^ in DDP and NS, respectively) or elevated eIF4E group (2843.25 ± 216.55 mm^3^, 3398.52 ± 323.22 mm^3^ in DDP and NS, respectively). Concurrently, repression of eIF4E contributed to boost the tumor growth inhibition of DDP, and tumors with eIF4E knockdown were much more sensitive to DDP than the blank control group (*P* = 0.0187, Figure 4D). In contrast, there was no statistical difference between DDP treated and NS treated animals on the effect of tumor growth inhibition in eIF4E overexpressed group, which indicated that increased eIF4E might invalidate DDP anticancer effect (*P* = 0.3034, Figure 4D).

### Overexpressed eIF4E promoted the PI3K/AKT signaling pathway and Bcl-2/Bax ratio, knockdowm of eIF4E aborgated this course in ESCC cells

**Figure 5:**
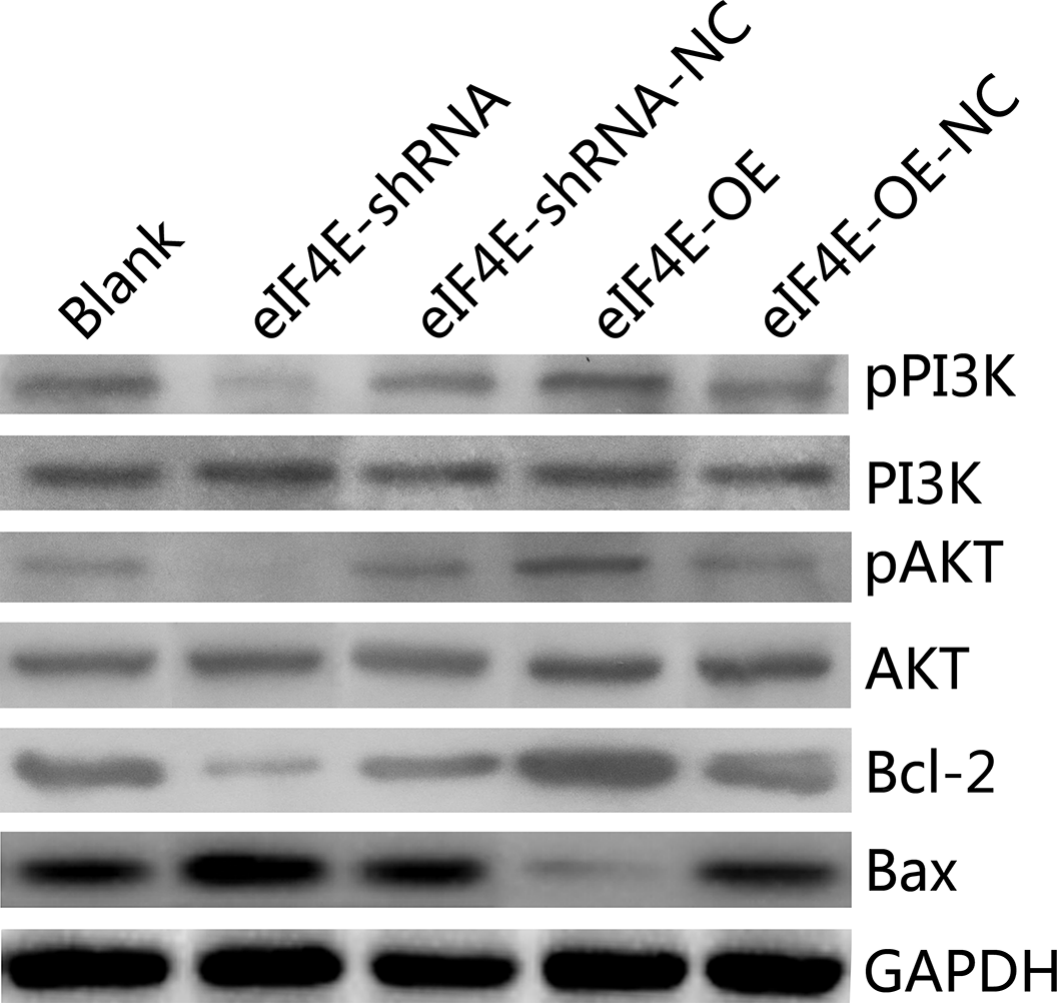
eIF4E promoted cisplatin-resistance in ESCC cells through the PI3K/AKT signaling pathway and increasing Bcl-2/Bax ratio In EC9706 cells with eIF4E inhibition, the protein levels of pPI3K, pAKT and Bcl-2/Bax ratio were significantly decreased, when compared to the negative control. Whereas they were significantly increased when eIF4E was overexpressed.

Plenty of studies have discovered the resistance to cisplatin in tumor cells both *in vivo* and *in vitro* [22, 23]. The PI3K/Akt pathway has been caught much of the attention for its increased pathway activation in resistant cancers, which also promotes abnormal expression of eIF4E [24, 25]. To explore whether eIF4E exerts its functions through the PI3K/AKT signaling pathways that contribute to cisplatin resistance, we examined a number of the main PI3K/AKT signaling pathway downstream target genes, including pPI3K, PI3K, pAKT, AKT, Bcl-2, Bax. PI3K/AKT played an important role in regulation of protein synthesis and cell growth. Bax which was regarded as an pro-apoptotic gene by increasing mitochondrial membrane permeability and initiating apoptosis, while Bcl-2 was one of the anti-apoptotic proteins, could antagonize Bax-induced apoptosis, the ratio of Bax/Bcl-2 represent the apoptotic activity. Expression of PI3K and AKT were inhibited in EC9706 cells that stably down-regulated eIF4E, as well as the Bcl-2/Bax ratio, with the Bax was promoted and the Bcl-2 was decreased (Figure 5, lane 2 and 3). Moreover, overexpression of eIF4E in EC9706 cells reversed the PI3K/AKT/Bax/Bcl-2 expression (Figure 5, lane 4 and 5). These data indicate that eIF4E promotes PI3K/AKT signaling pathway and Bcl-2/Bax ratio in EC9706, which may imply the possible mechanism of eIF4E induced cisplatin resistance in ESCC.

## DISCUSSION

Abundant researches have demonstrated that overexpressed eIF4E was significantly correlated with tumor malignant characteristics, resistance to therapy, and regarded as the poor prognosis predicator for human cancers [10, 14–17, 21, 26–28]. Targeting eIF4E for inhibition may provide an attractive therapy for many different tumor types [7, 10, 14, 18, 19]. Here, we present data to evaluate the potential therapeutic targeted role of eIF4E in ESCC. We demonstrated that eIF4E expression was significantly elevated in clinical ESCC tissues. High expression of eIF4F significantly promoted EC9706 cells proliferation, survival, transferance and invasion, which could be interpreted by the excessive function of weak mRNAs. Overexpressed eIF4E was closely associated with chemoresistance to cisplatin-based treatment both *in vitro* and *in vivo* models of ESCC. In contrast, knockdown of eIF4E expression showed a significant delay for tumorigenicity, as well as decrease in tumor size and tumor weight compared to the mice with control or overexpressed eIF4E. We also found that eIF4E knockdown significantly potentiated cisplatin - induced cytotoxicity and long-term cell growth suppression, hence sensitizing esophageal cancer cells to cisplatin treatment *in vitro* and *in vivo*.

As summarized in Table 1, we did not find any correlation between eIF4E expression and grade of ESCC as expected, but we found a strong association between eIF4e expression and lymphonodus involvement and TNM stage, which are tightly associated to tumor invasion and metastatic dissemination that manifested with shorter DFS and OS survival time, and multiple Cox regression analysis indicated that eIF4E was an independent unfavorable predicator for poor prognosis in ESCC. Although further validation may be necessary, these data suggest that high expression of eIF4E in tumor, together with traditional risk factors such as lymphonodus status and tumor staging, may serve as a biomarker for a metastatic phenotype and a prognostic factor for poor survival in human ESCC.

We found in this study that weak mRNAs including VEGF, FGF-2, MMP-9, Cyclin D1, Survivin and C-myc were positively regulated by eIF4E. With long or structured 5′UTRs, the translation of weak mRNAs are eIF4E-dependent and strictly regulated *in vivo*. Even a mild enhanced eIF4E level shows a marked potential to oncogenesis *in vitro* and *in vivo* [29]. Most of the “weak mRNAs” expression level in serum are also common predicators for poor prognosis owing to tumor progression and metastasis [30, 31]. For those properties and a positive correlation between eIF4E and weak mRNAs, we postulate that eIF4E involve in ESCC oncogenesis and progression through weak mRNAs promotion for the first time.

Cisplatin, docetaxel, and 5-FU regimen are recommended as category 1 treatment for esophageal carcinoma with distant metastasis, according to the guidelines of the National Comprehensive Cancer Network (NCCN) [32]. However, their application is limited by both intrinsic and acquired chemoresistance. Plenty of studies have discovered the resistance to cisplatin in tumor cells both *in vitro* and *in vivo* [30, 31], but the literature regarding eIF4E and ESCC chemosensitivity is scant. To our knowledge, this is the first report to investigate the possible role of eIF4E in improving chemosenstivity in ESCC. Our results demonstrated that inhibition of eIF4E expression resulted in an augmented cell viability inhibition *in vitro* and a significant tumor reduction and synergistic effects with cisplatin in a xenograft model. These findings supported previous studies by Oridate et al. [33] and Dong et al. [19], who reported that decreased eIF4E could synergize cisplatin to suppress tumor cell growth by enhancing chemosenstivity in head and neck carcinoma and breast cancer. However, the exact molecular mechanisms of how eIF4E contributes to chemoresistance remain disputable [18, 19]. It has been well known that the abnormal PI3K/AKT pathway was involved in the cisplatin resistance and ESCC cells [34, 35]. Moreover, abnormal eIF4E expression promoted rapamycin resistance by abberant activation of AKT [36]. In the present study, we have demonstrated that eIF4E induced cisplatin-resistance in ESCC mainly by promoting PI3K/AKT pathway and increasing Bcl-2/Bax ratio, which is similar to eIF4E in triple-negative breast cancer cells [18]. Moreover, we found that Survivin and FGF-2, which were responsible for the resistance to the pro-apoptotic effect induced by many chemotherapy drugs, were also inhibited. Moreover, as it shown in apoptosis analysis, knockdown of eIF4E increased apoptosis rate in ESCC cells significantly. All these indicated that eIF4E involved in cisplatin resistance in ESCC mainly via disrupting the balance of apoptosis.

Although inspiring, the present study still has several limitations: Firstly, it was mainly carried out in EC9706 cells. Considering that most cancer tumors are highly heterogeneous, we need to validate our findings in other ESCC cell lines. Secondly, even though a promissing prognosis predicting role of eIF4E was found in ESCC, further validation is required for the clinical application.

Taken together, our results showed that eIF4E overexpression correlated to ESCC poor prognosis and exerted an oncogenic role in ESCC. Overexpression or knockdown of eIF4E by RNA intervention modulated tumor cell growth and chemosensitivity to cisplatin both *in vitro* and *in vivo*, which suggested that eIF4E might have a great potency of synergistic effect of cisplatin to ameliorate drug resistance in ESCC and many other tumors. The combination of targeting eIF4E and traditional anti-cancer agents will be a novel strategy in targeted cancer therapy and may become a promising substitute for the conventional chemotherapy in ESCC.

## MATERIALS AND METHODS

### Ethical statement

Written informed consent was obtained from all participants, and the study protocol was approved by the ethics committee of Xiangya Hospital, Central South University (CSU). All mouse experiments were approved by the Animal Care and Use Committee and conducted in accordance with the official recommendations of the Care and Use Laboratory Animals of Xiangya Hospital, CSU.

### Clinical specimens and cell lines

90 tumor samples and paired adjacent non-cancerous tissues (ANCTs) were obtained from 90 ESCC patients subjected to esophagectomy in Xiangya Hospital of Central South University between July, 2010 and August, 2014, who were diagnosed with ESCC by more than two pathologists. ANCTs were collected more than 5 cm away from the margin of the tumor, and those normal esophageal tissues were obtained from more than 7 cm away from the tumor margin or from the normal individual who took routine gastroscopy. Patient clinical parameters were summarized in Table 1. Informed consent was obtained from each patient before participation in this study. The follow-up schedule of patients was every 3 months during the first postoperative year and at least 6 months afterward for survival and recurrence inquiry until death or until the end of the investigation. All 90 patients (74 male and 16 female) were younger than 75-year old, and were first-diagnosed cases without chemotherapy, radiotherapy or other treatments. This study has been approved by Xiangya Hospital of Central South University Ethics Committee, and the usage of the information and specimens collected has been handled and made anonymous according to the ethical and legal standards. Diagnosis of ESCC was performed by two specialized pathologists based on the World Health Organization (WHO) criteria. Tumor differentiation and staging was classified according to the 7th edition of TNM classification of UICC.

The subjects were followed-up every 3 months during the first postoperative year and at least 6 months afterward for survival and recurrence inquiry until death or until the end of the investigation.

Human ESCC cell lines (EC-1, EC109 and EC9706) were obtained from Chinese Academy of Science cell bank (Shanghai, China). A normal human esophageal epithelial cell line (*HEEpic*) was purchased from the American Type Culture Collection. Cells were cultured and maintained in RPMI 1640 supplemented with 10% fetal bovine serum (FBS), 100 U/ml penicillin and 100 ug/ml streptomycin in a humidified incubator with 5% CO_2_ at 37°C. All culture materials were purchased from GIBCO, USA.

### Quantitative real-time PCR (qRT-PCR)

Total RNA was extracted from tissues or cells using TRIzol reagent (Invitrogen, USA) and was stored at −20°C until use. Reverse Transcription System (Promega) was used for cDNA synthesis according to the protocol provided by manufacturer. The mRNA expression levels of eIF4E was measured by quantitative Real time-PCR using the ABI PRISM 7500 Sequence Detector System (Applied Biosystems, USA), and was normalized to an internal standard (glyceraldehyde-3-phosphate dehydrogenase, GAPDH). PCR primer used were as follows: eIF4E: forward, 5′-TGTGGCGCTGTTGTTAATGT-3′, reverse, 5′- GCGTGGGACTGATAACCAAT-3′; GAPDH: forward, 5′- AGACAGCCGCATCTTCTTGT-3′, reverse, 5′- TGATGGCAACAATGTCCACT-3′. The reaction protocol involved heating for 3 min at 95°C, followed by 45 cycles of amplification (15s at 95°C and 1min at 60°C). All reactions were done in triplicate using 20 μl samples containing 50 ng of complementary DNA. The eIF4E expression was analysed using the 2^−ΔΔCT^ method, as the eIF4E expression was identified high when the log_2_ (fold change) is more than 1.

### Immunohistochemistry staining

Slides were stained according to manufacturer's protocols for eIF4E proteins. In brief, paraffin-embedded sections (5 μm) were grilled at 65°C for 1h, then dewaxed in xylene and rehydrated in an increasing diluted ethanol series. High-temperature antigen retrieval included microwave-treatment in 0.1 M citrate solution (pH 6.0) for 10 min and 3% H_2_O_2_ incubated at room temperature for 20 min and goat serum incubated for 20 min at room temperature to block endogenous peroxidase activity, followed by incubation with diluted primary antibody (1:75) 4°C overnight. Next day the slides were treated with the secondary antibody for 20 min at room temperature, followed by DAB (diaminobezidin) colorimetric visualization. A semi-quantitative scoring system was used based on both the staining intensity and positive extent. Immunohistochemical staining was scored independently by two pathologists without knowledge of the patients' characteristics. Any discrepancy was resolved by taking the consensus view. The score of immunoreactivity was performed by calculating the extent and intensity of staining positivity of the cells in a semi-quantitative manner.

### Cell culture, plasmids construction, and cell transfection

### Cell culture

Human ESCC cell lines EC9706, EC109, EC-1, and *HEEpic* were obtained from Institute of Tumor Research, Chinese Academy of Medical Sciences. 293T cell was obtained from Shanghai JiKai Gene Chemical Technology (Shanghai). Cell lines were routinely maintained in RPMI (Gibco, Life Technologies, Waltham, MA, USA) containing 10% calf serum (Sijiqing Biological Engineering, Hangzhou, China) at 37°C in a humidified incubator under 5% CO_2_.

### Plasmid construction and cell transfection

To investigate endogenous eIF4E protein biological functions, we were tempted to regulate eIF4E expression level by RNA interference. A plasmid called eIF4E-PEGFP-N1 is constructed for eIF4E overexpression. According to the principle of siRNA synthesis, to knockdown eIF4E expression, 3 DNA template oligonucleotides corresponding to eIF4E gene (GenBank ID BC012611) were designed and synthesized by siRNA Target Finder as follows: eIF4E–shRNA1 (sense, 5′-CCA AAGATAGTGATTGGTTATCTCGAGATAACCAATCA CTATCTTTGG-3′), eIF4E-shRNA2 (sense, 5′-CGGCTG ATCTCCAAGTTTGATCTCGAGATCAAACTTGGAGA TCAGCCG-3′), eIF4E–shRNA3 (sense, 5′-CCGACTACA GAAGAGGAGAAACTCGAGTTTCTCCTCTTCTGTA GTCGG-3′), and a non-specific shRNA, NS (sense, 5′-GATCTAAGCATTAGGTACAGCATTTCAAGAGAATGCT GTACCTAATGCT-3′). All of the above sequences were inserted to construct U6-shRNA-CMV-GFP-vevtor, and were named as U6-eIF4E-shRNA1-CMV-GFP, U6-eIF4E-shRNA2-CMV-GFP, U6-eIF4E-shRNA3-CMV-GFP and U6-NS-CMV-GFP vector, respectively. Primers and siRNA oligoes used for cell transfection were individually synthesized by JiKai GENE Chemical Technology co., LTD, Shanghai. siRNA against eIF4E were individually transfected into EC9706 cells using Lipofectamine Plus (Grand Island, NY) according to the manufacturer's protocols, and were selected with 200 μg/ml of G418 (Sigma, USA) for 14 days. The transfected single clones were isolated and expanded for an additional two months in media containing 200 μg/ml of G418, cultured at 37°C in 5% CO_2_ and saturated humidity. The eIF4E protein and mRNA of those transfected cells were detected by WB and qRT-PCR to confirm the transfection efficiency. The cells transfected with U6-eIF4E-shRNA2-CMV-GFP was discontinued for subsequent assays because of knockdown of eIF4E expression with the maximum efficiency.

### Western blot analysis

Western blotting was performed as described elsewhere [20]. Briefly, EC9706 cells or tumor tissues were collected and lysed on ice. The extracts were centrifuged at 12,000 g for 20 min and the concentration of supernatant fractions was measured. The protein samples were denatured by boiling for 10 min and loaded onto SDS–PAGE (10%) gel for electrophoresis and transferred onto PVDF membrane (Millipore, USA). Membranes were immunoblotted with primary antibodies or GAPDH at 4°C overnight. Signals were detected by enhanced chemiluminescence (ECL; Amersham). The levels of eIF4E protein expression were determined densitometrically and normalized to GAPDH.

### Colony formation assay, transwell migration and invasion assay, flow cytometry assay for apoptosis

For colony formation assay, cells were counted and seeded (800 cells/well) in culture dish (10 cm) (in triplicate). Fresh culture medium was replaced every 3 days. Colonies were counted only if they contained more than 50 cells, and the number of colonies was counted at 14 days after seeding. The cells were stained using Giemsa. The ability of colony formation was calculated by the colony formation number.

The migration and invasion assays were carried out using Transwell insert chambers (Corning, USA). For migration assay, after starvation for 24 hours, 4 × 10^4^ cells were plated into the upper chamber in serum-free medium in triplicate. Medium containing 5% FBS in the lower chamber served as chemoattractant. After incubation for 24 h at 37°C in a 5% CO_2_ humidified incubator, cells in the upper chambers were removed by wiping with a cotton swab and cells migrated to the lower surface of filter were fixed in 70% ethanol for 30 min and stained with 0.2% crystal violet for 20 min. Cell migration was scored by counting ten random fields per filter under a light microscope. For invasion assay, after starvation for 24 hours, 4 × 10^4^ cells were seeded into upper chambers precoated with matrigel (Corning, USA) in serum-free medium in triplicate. Medium with 5% FBS were added to the lower chamber to serve as chemoattractant. After incubation for 48 h at 37°C, non-invading cells on the upper surface of filter were removed with cotton swabs and invading cells that migrated to the lower surface of filter were fixed, stained and scored as described above.

Apoptosis was analyzed by flow cytometry assay. 3 × 10^5^ cells were harvested at 48 h post-transfection per well, and fixed in 70% ethanol overnight at 4°C. The cells were stained with Annexin V-FITC and propidium iodide. A total of 30 000 cells were sorted by FACSCalibur System and cell cycle profiles were analyzed using the Flowjo software. Apoptosis was determined by dual staining with Annexin V-FITC/PI. The relative proportion of Annexin V-positive cells was determined using the Flowjo software and counted as apoptotic cells. The assays were carried out in triplicate for three times.

### *In vitro* chemosensitivity assay

Cell viability was measured by a 3- (4,5-dimethyl-2-thiazolyl)- 2,5-diphenyl -2 -H-tetrazolium bromide (MTT) assay. eIF4E or negative control shRNA-transfected EC9706 cells were seeded into 96-well plates (4 × 10^5^/well) in culture medium for 24 h. The medium was then changed to a medium containing the following concentration of DDP (cisplatin) at 0, 6.0, 12.0, 24.0, and 48.0 μg/ml. The cell viability was assessed at 24 h and 48 h incubation. Every well was stained with 5 mg/ml MTT for 4 h and lysed for 15 min. Then the optical density (OD) of culture plates was determined on a microreader (Bio-Rad) at 570 nm. Inhibitory rate was calculated as (1- OD 570_treated group_/OD 570_untreated group_) *100%. Each experiment was repeated three times.

### Tumor xenograft models and chemosensitivity to cisplatin *in vivo*

Thirty six male BALB/C nude mice (SPF, 4 weeks of age, 20–22 g) were purchased from Slac Laboratory Animal, Chinese Academy of Sciences (Shanghai, China) and were raised under specific pathogen-free conditions at Central South University Laboratory Animal Division. All forms of surgery were performed under anesthesia with sodium pentobarbital. Nude mice were divided into 3 groups (*n* = 12 for each) and were subcutaneously (*s.c.*) inoculated into the right flanks with 3 × 10^6^ of eIF4E-transfected or scramble control-transfected EC9706 cells (eIF4E-PEGFP-N1 for eIF4E overexpression, eIF4E-shRNA for eIF4E knockdown, and EC9706 cell only for blank control), respectively. To ensure a consistent size at the outset of treatment, tumors were measured with calipers daily once they reached to the average volume of 100–200 mm^3^. Tumor bearing mice in each group were allocated randomly into two sub-groups: the treatment group of 6 mice were administered with cisplatin (DDP, 2 mg/kg; i.p., twice a week for 4 weeks), and the control group of 6 mice were injected with normal saline (NS) with identical amount and procedure. Treated and control tumors were measured weekly during therapy. Mice were sacrificed after 4 weeks of cisplatin injection, and tumors were taken out and excised. Tumor volume was calculated by the formula: 0.5 × L × W^2^ (L = length of tumor; W = width of tumor).

### Statistical analysis

GraphPad Prism 5.0 software was used for statistical analyses. All data are presented as Means ± standard deviations (SD). Categorical variables were compared by χ2 test. Continuous variables were compared using independent two sample *t-test*. Multivariate analyses were performed by the Cox proportional hazard model. Survival curves were done by the Kaplan–Meier method (the log-rank test). All tests were two-tailed and a *P* < 0.05 was considered to be statistically significant.

## SUPPLEMENTARY MATERIAL FIGURES


