# Supplementary material for: eIF4E promotes tumorigenesis and modulates chemosensitivity to cisplatin in esophageal squamous cell carcinoma
Source: Oncotarget. 2016 Aug 30;7(41):66851–64. doi: 10.18632/oncotarget.11694 (PMC5341842; doi:10.18632/oncotarget.11694)
Supplement: Supplementary file 1 [file oncotarget-07-66851-s001.pdf]

eIF4E promotes tumorigenesis and modulates chemosensitivity to cisplatin in esophageal squamous cell carcinoma

Supplementary Materials

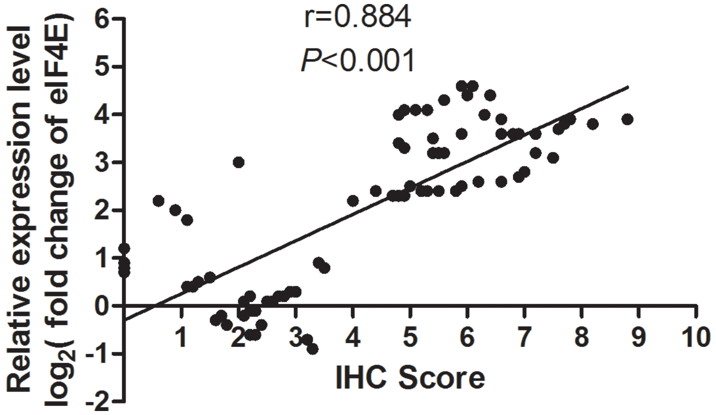

Supplementary Figure S1: eIF4E protein expression was positively associated with eIF4E mRNA expression in clinical ESCC tissues. eIF4E protein expression was evaluated by IHC score, eIF4E mRNA expression was expressed as $\log_2$ (fold change). Scatter plot shows a strong positive correlation between them (n = 90).

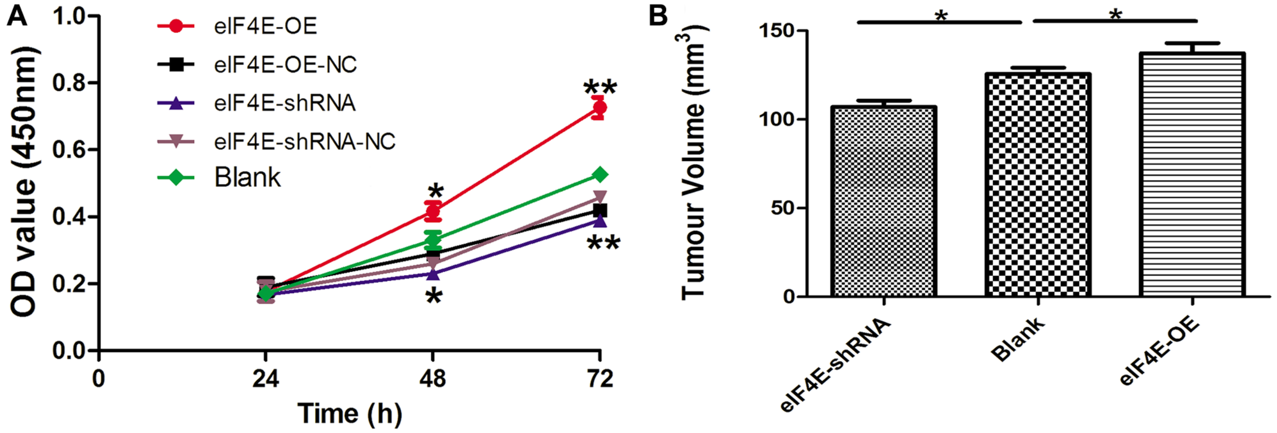

Supplementary Figure S2: eIF4E promoted tumorigenesis *in vitro* and *vivo*. (**A**) Knockdown of eIF4E reduced cell in ESCC cells,while overexpressed eIF4E promoted cell proliferation. Cell proliferation was determined by MTT assays. (**B**) Overexpression of eIF4E promoted tumorigenicity in *vivo* when compared to Blank control mice, while knockdown of eIF4E significantly decreased the primary tumor size. Error bars indicate s.d. (n = 3). *P < 0.05; **P < 0.01.

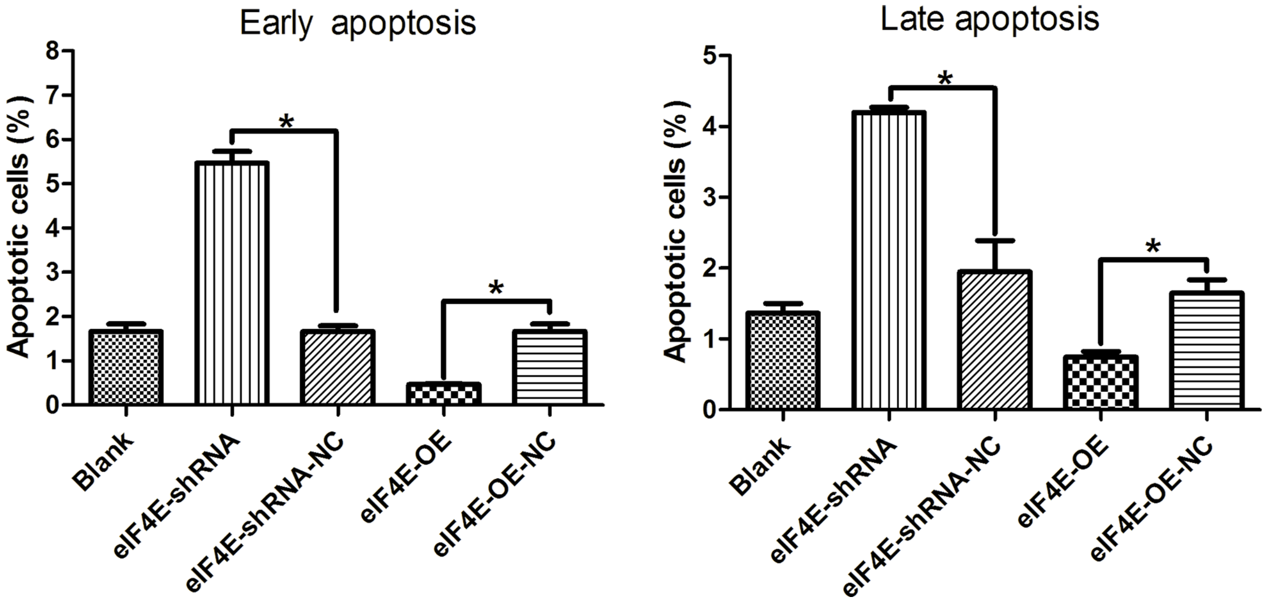

Supplementary Figure S3: eIF4E inhibited both early apoptosis and late apoptosis. Statistic analysis of data in flow cytometry assay with double staining by Annexin V-FITC/Propidium Iodide, Error bars indicate s.d. ($n = 3$). *$P < 0.05$.

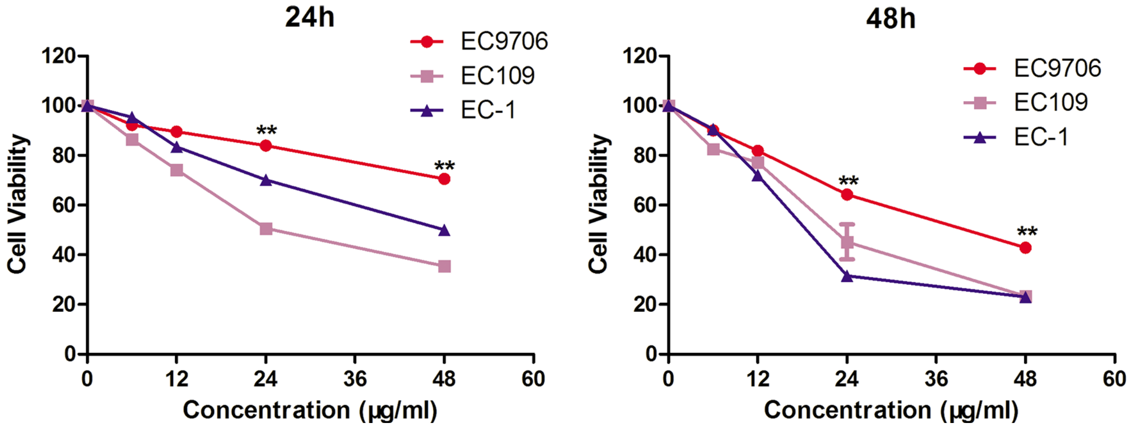

Supplementary Figure S4: Different ESCC cell lines exhibited a distinct sensitivity to DDP. After treated with DDP (cisplatin, 0–48 μg/ml) for 24 h and 48 h, the cell viability of EC9706, EC-1 and EC109 cells were detected by MTT, respectively. The plots show that inhibition of cell survival in a time and concentration dependent manner, EC9706 cells showed a significant resistance to DDP, when compared to EC-1 and EC109 cells. Error bars indicate s.d. ($n = 3$). **$P < 0.01$.
